# Supplementary material for: Chromothripsis during telomere crisis is independent of NHEJ, and consistent with a replicative origin
Source: Genome Res. 2019 May;29(5):737–49. doi: 10.1101/gr.240705.118 (PMC6499312; doi:10.1101/gr.240705.118)
Supplement: Supplemental Material [file supp_gr.240705.118_Supplemental_file_1.zip › contigs/annotated_contigs/DB113/contig.3.DB113_length_771_mean_cov_11.1893644617.docx]

**DB113_length_771_mean_cov_11.1893644617**

```
GTCACCACCAGGCCTGCCTTACAAGACCTCCTGAAGGAAGCGCTAAACATGGAAAGGAACAACCGGTACCAGCCTCTGCAAAATCATGC
 >chr4:150838502-150838738 + E=5e-100
CAAAATGTAAAGACCATCGACACTAGGAAGAAATTGCATCAACTAACGAGCAAAATGACCAGCTAACATCATAATGACAGGATCAAATT

CACACACAACAATATCAGCCTTAAATGTAAAAGGACAAAATGTCCCAATTAAAAGACA|GAACAG|GGCTGGGCGAGGTGGTTCACGCC
                                                                 >chr7:7806933-7807055
TGTAATATCAGCACTTTGGGAGACTGAGGCAGGTGGATCATGAGGTCAGGAGATCGAGACCATTCTGGCCAATATGGTGAAACCCCATG
- E=2e-31 p=2e-02
TCTACTAAAA|TATA|TACAAAGAGACTTAGACTTCCACATAATAATATTGGGAGACTTTAACACCCCACTGTCAATATTAGATAGATT
               >chr13:85270598-85271001 + E=1e-224
ATCAAGACAGAAAATTAACAAAGATATTCAGGACTTGATCTCAGCTCTGGATCAAGTGGACCTGATAGATATCTGCAGAACTGTCCACC

CAAAAACAGCAGGATATAAATTCTTATTGGTGCCACATGGCACTTACTCCAAACTGATCACATAATTGGAAGTAATACACTCTTCAGCA

AATGCAAAAGAATTTAAATCATAACAAACAGTCTCTCAGACCACAGCACAATAAAATTAGAACTCAAGATTAAGAAACTCACTCAAAAC

CACACAACTACATGGAAATTGAACAACCTGCTTCTGAAGGACTCCTGGGTAAATAATGAAATT
```
